# English as a Foreign Language Learners’ Academic Achievement: Does Creativity and Self-Efficacy Matter?

**DOI:** 10.3389/fpsyg.2022.877679

**Published:** 2022-04-06

**Authors:** Shanshan Wang, Yaling Chen, Yi Wan

**Affiliations:** School of Media and Communication, Shanghai Jiao Tong University, Shanghai, China

**Keywords:** creativity, academic achievement, self-efficacy, EFL learners, education

## Abstract

Given the centrality of learners’ individual differences in their academic achievement, a plethora of research has been done on various individual difference variables and their associations with learners’ increased achievement. Yet, the role of creativity and self-efficacy in EFL learners’ academic achievement has not been extensively studied. Further, no review inquiry has simultaneously probed into the consequences of these two individual difference variables for learners’ academic achievement. This study strives to fill this underappreciated gap by addressing the positive consequences of creativity and self-efficacy for EFL learners’ academic achievement. The desirable consequences of creativity and self-efficacy beliefs were clarified using the available evidence. Finally, the conclusions and consequences of the findings are discussed.

## Introduction

The primary objective of any educational system is to lead pupils to academic success. That is, all educational practitioners, including administrators and instructors, strive to guide learners toward high levels of academic achievement. Academic achievement is “the outcome of learning, which is typically measured by classroom grades, classroom assessments, and external achievement tests” (Gajda et al., 2017, p. 271). Put it another way, academic achievement refers to the amount of knowledge acquired by learners in a particular course (Winne and Nesbit, 2010). In the domain of language education, the concept of academic achievement has to do with the overall attainment of learners in a language course (Jin and Zhang, 2018). Given the fact that language learners’ individual differences can make a significant difference in their level of academic achievement (Schmidt, 2012; Dörnyei, 2014), several academics have probed into the role of individual difference variables (i.e., self-concept, self-esteem autonomy, emotional intelligence, and motivation) in English as a Foreign Language (EFL) learners’ academic achievement (e.g., Marsh and Martin, 2011; Feng et al., 2013; Rastegar and Karami, 2013; Shao et al., 2013; Soodmand Afshar et al., 2014; Wang, 2017; Taheri et al., 2019; Li, 2020; Lin, 2021, to cite a few). Unlike other individual difference variables, creativity and self-efficacy have not been widely studied.

Creativity as a prime example of individual difference variables generally refers to “the potential of persons to produce creative works whether or not they have produced any work as yet” (Glover et al., 2013, p. 3). In the realm of education, learner creativity pertains to the ability of learners to create or bring into existence something innovative and novel, whether an innovative solution to an educational challenge or a novel strategy for learning educational content (Wang, 2018). On the importance of creativity in education, Pishghadam et al. (2011) postulated that creative learners are more likely to attain desirable learning outcomes. It is partially due to the fact that, in the absence of creativity, learners are forced to rely on old and obsolete learning strategies that are ineffective for acquiring new knowledge (Sandri, 2013). In this regard, also noted that creative learners commonly employ novel and innovative strategies that help them achieve higher academic grades (Fang et al., 2016).

Self-efficacy, as another individual difference variable, pertains to “one’s beliefs, thoughts, and perceptions about his/her personal abilities and capabilities” (Bandura, 1994, p. 72). Likewise, learner self-efficacy refers to individual learners’ ideas and viewpoints about their capability to learn and master the course content (Cotterall, 1999). Applied to the language education domain, learners’ self-efficacy deals with how positively they perceive their own capacity and ability to acquire a new language. In fact, language learners’ self-efficacy has to do with the extent to which they believe in their language learning competence and skills (Raoofi et al., 2012; Han and Wang, 2021). As put forward by Tilfarlioglu and Cinkara (2009, p. 130), “self-efficacy beliefs influence human functioning through cognitive, motivational, affective, and decisional processes”. Relying on this statement, Nwosu and Okoye (2014) submitted that language learners’ academic performance may be remarkably influenced by their self-efficacy beliefs. In a similar vein, Hwang et al. (2016) declared that learners with higher levels of self-efficacy commonly outperform other learners.

Due to the value of creativity and self-efficacy in achieving higher academic outcomes (Fang et al., 2016; Hwang et al., 2016), several investigations have been performed in general education to explore the effects of these individual difference variables on learners’ academic achievement (e.g., Olatoye et al., 2010; Alivernini and Lucidi, 2011; Nami et al., 2014; Atwood and Pretz, 2016; Llorca et al., 2017; Olivier et al., 2019, to cite a few). Yet, the impact of EFL learners’ creativity and self-efficacy on their academic achievement has not been extensively researched (Pishghadam et al., 2011; Rezaei, 2012; Asakereh and Yousofi, 2018; Zokaee et al., 2020). Moreover, no review inquiry has simultaneously investigated the consequences of these two individual difference variables for EFL learners’ academic achievement. The current study strives to bridge the lacuna in the literature by scrutinizing the influence of creativity and self-efficacy on EFL learners’ academic achievement.

### Learner Creativity

The term “creativity” has been generally conceptualized as “the interaction among aptitude, process, and environment by which an individual or group produces a perceptual product that is both novel and useful” (Plucker et al., 2004, p. 92). As a multifaceted variable, creativity encompasses two major components (Trevlas et al., 2003): *novelty* and *relevance*. The first component, novelty, pertains to the emergence of a new and innovative idea in the human mind (Gruys et al., 2011). The second component, relevance, signifies that creativity always happens in a particular context and “a creative act is a response to a situation in which something requires a solution or at least clarification” (Hajilou et al., 2012, p. 132). According to Agars et al. (2008), creativity is the employment of inspiration, imagination, and unique ideas to reach a certain objective. Extending this definition to the context of education, Lee (2013) described learners’ creativity as the utilization of unique and innovative ideas by individual learners to fully understand the course content.

### Learner Self-Efficacy

As a psychological construct, self-efficacy pertains to one’s personal beliefs, perceptions, and attitudes toward his/her own potential, capacity, or capability (Zimmerman et al., 1992). In the same vein, learners’ self-efficacy relates to individual learners’ thoughts and conceptions of their academic capabilities (Zimmerman, 2000; Lane et al., 2004). Put simply, it deals with the degree to which learners have faith in their learning abilities (Schunk and Mullen, 2012). According to Klassen and Usher (2010), those learners who firmly believe in their academic capabilities are able to pursue the learning process despite the existing challenges, difficulties, and obstacles. To underline the value of self-efficacy beliefs, Galyon et al. (2012) mentioned that learners’ self-efficacy beliefs motivate them to put forth more effort in accomplishing their academic tasks. This, in turn, empowers learners to attain higher academic achievements (Caprara et al., 2011).

### The Role of EFL Learners’ Creativity and Self-Efficacy in Their Academic Achievement

To elucidate the role of creativity in raising EFL learners’ academic achievement, Zhang et al. (2020) stated that creative language learners typically adopt novel and innovative ways of learning that help them acquire a new language successfully. In a similar sense, Cassidy (2012) proposed creativity as a source of explanation for why some language learners attain higher academic outcomes. To him, increased academic achievement is tied with learners’ creativity as “desirable learning outcomes require divergent and reproductive ability” (Naderi et al., 2009, p. 102). Besides, to clarify the role of self-efficacy in improving language achievement, Wang and Neihart (2015) noted that self-efficacy belief as a motivational factor drives language learners to do their best to learn a new language.

## Empirical Studies

To date, some scholars have studied the role of EFL learners’ creativity and self-efficacy in their academic achievement (e.g., Nasrollahi and Barjasteh, 2013; Ghorbandordinejad and Afshar, 2017; Zokaee et al., 2020; Ozer and Akçayoğlu, 2021). Ghorbandordinejad and Afshar (2017), for instance, examined whether Iranian EFL learners’ academic achievement is subjected to their self-efficacy beliefs. To do so, two reliable scales were handed out among 400 Iranian EFL learners. Evaluating the correlation of the questionnaires, they discovered that Iranian EFL learners’ self-efficacy is highly correlated with their academic achievement. Moreover, self-efficacy beliefs were found to be a strong predictor of learners’ academic achievement. In another study, Zokaee et al. (2020) assessed the impact of EFL learners’ creativity on their academic achievement. To this end, two self-report questionnaires were given to 138 EFL learners. Inspecting the association of the questionnaires, they found a favorable relationship between learners’ creativity and their academic achievement. They also discovered that learners’ academic achievement can been largely affected by their creativity.

## Conclusion and Implications

So far, the literal and practical definitions of learner creativity, learner self-efficacy, and academic achievement were provided. Additionally, the role of EFL learners’ creativity and self-efficacy in their academic achievement was elucidated using theoretical and empirical evidence. Building upon the available evidence, one can reasonably infer that EFL learners’ academic achievement can be noticeably enhanced by their creativity and self-efficacy. The outcomes of this review study appear to be instructive and insightful for all English language learners in any EFL country. With respect to the value of creativity in raising learners’ academic achievement (Cassidy, 2012; Zhang et al., 2020), EFL learners need to make use of creative thinking to attain better academic outcomes. In addition, considering the positive effects of self-efficacy beliefs on learners’ achievement (Wang and Neihart, 2015), EFL learners should trust themselves and their academic abilities to improve their language achievements.

## Author Contributions

All authors listed have made a substantial, direct, and intellectual contribution to the work and approved it for publication.

## Funding

This work was supported by the National Social Science Fund of China (Grant No. 21AZD085). The research on Historical Experience of Centenary of the Communist Party of China’s Youth Publicity Work and Political Guidance of Youth in New Era.

## Conflict of Interest

The authors declare that the research was conducted in the absence of any commercial or financial relationships that could be construed as a potential conflict of interest.

## Publisher’s Note

All claims expressed in this article are solely those of the authors and do not necessarily represent those of their affiliated organizations, or those of the publisher, the editors and the reviewers. Any product that may be evaluated in this article, or claim that may be made by its manufacturer, is not guaranteed or endorsed by the publisher.
